# Electroacupuncture and Moxibustion Improved Anxiety Behavior in DSS-Induced Colitis Mice

**DOI:** 10.1155/2019/2345890

**Published:** 2019-02-07

**Authors:** Daneng Wei, Na Zhao, Lushuang Xie, Biao Huang, Zhiqi Zhuang, Yong Tang, Shuguang Yu, Qiaofeng Wu

**Affiliations:** ^1^Acupuncture and Tuina School, Chengdu University of Traditional Chinese Medicine, Chengdu, Sichuan 610075, China; ^2^Pharmacy School, Chengdu University of Traditional Chinese Medicine, Chengdu, Sichuan 610075, China

## Abstract

**Background and Aims:**

Psychological disorders are prevalent in patients with inflammatory bowel disease, but the underlying mechanisms remain unknown. The aim of this study was to study whether electroacupuncture (EA) and moxibustion (MB) can improve anxiety behavior in DSS-induced colitis mice and to investigate whether this effect is related to hypothalamic-pituitary-adrenocortical (HPA) axis.

**Methods:**

The colitis model was established by drinking 2.5% dextran sodium sulfate (DSS). DSS-induced colitis mice were treated by EA or MB. Disease activity index (DAI) was scored; intestinal morphological and pathological structure was observed; anxiety behavior was tested by the elevated plus maze and open field. The concentration of corticotropin-releasing hormone (CRH) and cortisol (CORT) in serum was measured by enzyme-linked immunosorbent assay (ELISA). The protein expression of CRH in the colon and hypothalamus was detected by Western blot (WB).

**Results:**

Both EA and MB treatments can improvethe morphology of their distal colonic mucosal epithelia, as well as the disease activity index. Meanwhile, anxiety behavior in colitis mice was improved slightly after EA and MB treatment. In addition, the levels of CRH and CORT in the serum were slightly improved after EA and MB treatment. These effects are further supported by WB results. The expression of CRH in the colon and hypothalamus was increased significantly after treatment, compared with the model group.

**Conclusion:**

EA and MB were able to regulate the concentration of CRH in serum and protein expression in the peripheral and central at different levels and promote the recovery of the HPA axis that may be the basis for EA and MB to improve colonic pathology and alleviate anxiety behavior in DSS-induced colitis.

## 1. Introduction

Ulcerative colitis (UC) is a common kind of chronic colonic inflammation disease in North America and Northern Europe with incidence varying from nine to twenty cases per one hundred thousand person-years [1]. In recent years, the incidence of UC has increased in developing countries such as China [2]. Therefore, the treatment of UC is very critical worldwide. A complex set of neural, epigenetic, and environmental factors lead to severe intestinal disease and long-term discomfort to the patients, sometimes throughout their entire life [3, 4]. The chronic clinical course often results in a reduced quality of life [5].

It has been recognized that psychological status and physiological stimuli play an important role in the development of functional gastrointestinal (GI) disorders such as inflammatory bowel disease (IBD) which comprises two major gastrointestinal disorders, namely, Crohn's disease (CD) and UC [6]. Depression has been projected to be the second leading cause of disability worldwide by 2020 [7]. Individuals with IBD experience 3 times the rate of depression compared with the general population. Over their lifetime, depression may affect more than 25% and anxiety more than 30% of individuals with UC [8–10]. A systematic review analysis, based on 79 primary studies, estimates that about 15% of patients with UC have depression and over 20% of patients have symptoms of depression, which is higher than expected in the general population worldwide [11]. There is a bidirectionality between psychological comorbidity (anxiety) and UC with each influencing the course of the other when they coexist [12]. These two psychiatric disorders might indeed predispose people to UC, or conversely, UC might predispose people to depression or anxiety seriously affecting the patient's life quality and the disease progression [13]. It is suggested that anxiety may exert a negative influence on the pathogenetic process of UC [14]. Therefore, the clinical treatment of UC should not only focus on reducing the patient's physical symptoms but also actively interfere with the patient's anxiety as well as other mental and emotional abnormalities, especially during periods of active disease.

In conjunction with clinical and animal experiment findings, a growing literature explores on how neuroendocrine mediates the relationship between UC and anxiety [15]. The best described neuroendocrine network is the hypothalamic-pituitary-adrenal (HPA) axis, a pathway regulating hypothalamic secretion of corticotrophin-releasing hormone (CRH), anterior pituitary secretion of adrenocorticotrophic hormone (ACTH), and adrenal cortical secretion of cortisol, which then provides feedback at the hypothalamus and pituitary (as well as other brain structures such as the hippocampus) and has downstream effects on other organs and physiologic system [16, 17]. Several studies have suggested that CRH plays a vital role in the occurrence of anxiety, as a major transmitter for stress response in the HPA axis [18]. Hyperactivity of the HPA axis in an anxiety state may result from hypersecretion of CRH [19–21]. However, few studies to date implicate the CRH or their receptors alleviating anxiety behavior in DSS-induced colitis.

There is currently a lack of therapeutic measures that can effectively prevent anxiety associated with UC, and it is urgent to find a safe and effective new treatment for UC. Acupuncture and moxibustion as an alternative and complementary therapeutic interventions have been used in China and some countries as alternative treatments for gastrointestinal disease [22]. Various reports have demonstrated that both EA and MB can effectively improve damaged intestinal tissues owing to inflammation [23] and suppress the inflammatory response of UC [24].

According to clinical and animal studies, acupuncture is widely used in the treatment of emotional disorders such as anxiety [25–29]. For animal studies, acupuncture was effective in improving sucrose consumption, latency, and food intake in chronic unpredictable mild stressed rats [29]. Acupuncture at acupoint ST36 (Zusanli) attenuates chronic stress-induced depression-like symptoms by modulating the HPA axis [30]. For clinical studies, many studies discussed the efficacy of acupuncture in treating and relieving the symptoms of depression [31]. In addition, a meta-analysis with the results from relevant randomized clinical trials supported that acupuncture was an effective treatment that could significantly reduce the severity of disease in the patients with depression [32]. In recent years, an increasing number of people are beginning to pay attention to interfere with the UC patient's anxiety as well as other mental and emotional abnormalities. However, whether EA and MB may improve anxiety associated with UC remains unknown.

Therefore, in this study, we will design the anxiety behavior in a DSS-induced colitis mouse study so as to attest if EA and MB can ameliorate anxiety associated with UC and whether their effects are obtained from improving the balance of the HPA axis. In addition, we will also investigate the CRH center target of HPA axis-related mechanisms.

## 2. Methods

### 2.1. Ethics Statement

All experimental procedures were approved by the Animal Care and Use Committee of Chengdu University of Traditional Chinese Medicine.

### 2.2. Animals and UC Model Inducing

**Figure 1:**
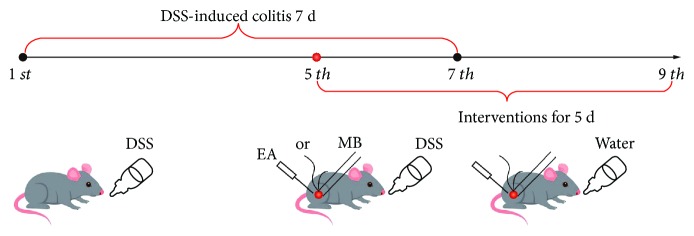
Schematic diagram illustrating the major steps of the experimental procedure in the dextran sodium sulfate- (DSS-) induced colitis model and EA or MB inventions. The MD, EA, and MB mice were induced to the ulcerative colitis model by drinking 2.5% DSS for 7 d. After establishment of the model on the 5^th^ day, the EA and MB groups were treated by electroacupuncture or moxibustion, respectively, for 5 d. NG: normal group; MD: model group; EA: electroacupuncture group; MB: moxibustion group.

All experimental animals were purchased from the Sichuan Dashuo Experimental Animal Co. Ltd. (license number: SCXK (Chuan) 2015-030). Male Kunming mice (25 ± 2 g) were housed in an environmentally controlled vivarium under a 12 : 12 h light-dark cycle starting at 8 AM (temperature 20 ± 2°C, humidity 50–60%) and had free access to diet and drinking water. After 1-week adaptation, the mice were randomly divided into the normal group (NG), model group (MD), electroacupuncture group (EA), and moxibustion group (MB) (*n* = 12/group). The MD, EA, and MB mice were induced to the ulcerative colitis model by drinking 2.5% dextran sodium sulfate (DSS, 43 kDa, MP Biomedicals) (Figure 1) [23].

### 2.3. EA or MB Interventions

After the establishment of the model on the 5^th^ day, “Guanyuan” (CV4) and “Zusanli” (ST36) were, respectively, selected for EA and MB treatments. Acupoints CV4 and ST36 were considered to be effective in treating UC according to clinic practice and our previous studies [33]. ST36 was alternately performed at the left or right lower limbs. The locations for these acupoints were determined according to Government Channel and Points Standard GB12346-90 of China and “The Veterinary Acupuncture of China.” For the EA group, EA was performed at the ST36 and CV4 points of the mice, with a 0.25 mm needle with a length of 13 mm being introduced to a depth of 3 mm. The acupuncture procedure was manipulated with a connected Hans-200 acupoint nerve stimulator to provide a sparse dense wave at a frequency of 2/15 Hz for 15 min once daily for 5 d. For MB, the mice were fastened on the special fixer (self-designed) with a supine position. The moxa stick (diameter × length: 0.4 × 10 cm, Nanyang Hanyi Moxibustion Technology Development Co. Ltd., China) was burned to carry out moxibustion over the ST36 and CV4 for 15 min. Mice in the NG and MD groups without EA or MB intervention were restricted for 15 min (Figure 1).

### 2.4. Anxiety Behavior

We used two tests of anxiety behavior: the elevated plus maze (EPM) and open field test (OFT). All tests were conducted during the dark cycle, beginning 30 min after lights were turned off and after an acclimation period of at least 1 h to the testing room. The testing room was dimly lit by a red lamp with luminosity between 5 and 20 luxes.

#### 2.4.1. EPM

At the beginning of the EPM test, the mice were placed in the central area facing one of the open arms. The behavior during the 5 min test period and the number of entries into the open and closed arms were scored, and the duration of time spent in each type of arm was measured [34, 35]. All activities were scored using an automated video-tracking system (Ethovision XT 9, Noldus). Additional parameters determined in data analysis were open-arm entries (OE), open-arm time (OT), and the percentage of open-arm entries (OE%) and open-arm time (OT%).

#### 2.4.2. OFT

The open field apparatus was constructed of black plywood and measured 55 × 55 cm with 50 cm walls. A central zone (20 × 20 cm) was drawn in the middle of the open field [36]. Mice were put on the central zone, the video camera installed on the top of the open field apparatus was started to record the locomotor of mice at the same time. Each mouse was individually placed in the central zone and recorded for 5 min. The number of visits to the central zone as well as the time spent in this central zone was scored. Between the intervals of two tests, the open field was cleaned with clothes dampened with 50% ethanol. Each animal was determined after modeling, as well as after EA or MB separately [37, 38].

### 2.5. General Assessment of Colitis

The severity of colitis and therapeutic effect were assessed by general manifestation, occult blood, and disease activity index (DAI). General manifestation includes weight, complexion, psychomotility, and fecal appearance (Table 1). The DAI is expressed as the equation: DAI = (body weight loss + characteristics of feces + fecal occult blood)/3 [23]; DAI was calculated three times weekly.

### 2.6. Sample Collection

Following the last experimental procedure, mice in each group were fasted for 24 h but allowed free access to water. Mice were anesthetized with 1% pentobarbital sodium at a concentration of 3 mL/kg. Then all mice were killed by cervical dislocation. At necropsy, the brains were removed quickly, and the hippocampus was isolated and stored at −80°C or 4% paraformaldehyde solution for the next process. At the same time, the colon was removed, cut open, cleaned with normal saline, measured, and photographed using a mobile phone. Then, all of the colonic tissues were divided into two parts: one segment was immersed and fixed in a 4% paraformaldehyde solution and stored at 4°C for hematoxylin-eosin staining. While the other segment was frozen at −80°C for further Western blot assay.

### 2.7. Histopathological Observation

The corresponding intestinal tissues stored at 4°C were taken and dehydrated in an alcohol series. A paraffin-embedded colon was cut into sections (thickness: 3–5 *μ*m), the sections were deparaffinized by xylene, and xylene was removed by alcohol. Then tissue sections were stained with hematoxylin and eosin (HE) and the morphology of distal colonic mucosal epithelia under the electron microscopy were observed (Mike Audi BA200 Digital).

### 2.8. ELISA Assay

The levels of CRH and CORT in the serum were detected by enzyme-linked immune absorbent assay (ELISA). All of the reagents and samples were prepared at room temperature before use, and the samples were centrifuged again after thawing before the assay. The method of the test was in compliance with the manufacturer's protocol.

### 2.9. Western Blot Analysis

Western blot analysis was performed as follows [39]: the brain tissues (in paraventricular nucleus (PVN)) and distal colon tissues of mice were homogenized on ice using a lysis buffer supplemented with ethylenediaminetetraacetic acid- (EDTA-) free complete protease inhibitors for protein extraction. The supernatant was collected following centrifugation at 12000 rpm/min for 10 min at 4°C. The protein concentration was measured using bicinchoninic acid (BCA) assay. Then the proteins of each sample were denatured at 95°C for 5–10 min and fractionated through 12% SDS polyacrylamide gel electrophoresis (PAGE) on a 10% gel. The bands were transferred to a polyvinylidene difluoride membrane. The blotted film was blocked for 1 h at room temperature in blocking solution (1 × TBS with 5% nonfat milk and 0.1% Tween 20) placed on the shake. The films were incubated overnight at 4°C with a primary antibody: CRH (Abcam Inc., USA) used at 1 : 200 dilution, then washed three times with TBST (1 × TBS with 0.01% Tween 20). The films were then incubated with a secondary antibody at room temperature for 1 hand then washed three times with TBST. Normalization was performed by blotting the same film with the anti-*β*-actin antibody (Abcam Inc., USA). All Western blot data were analyzed by Image-Pro Plus 6.0 software.

### 2.10. Statistical Analysis

All data are expressed as the mean ± standard deviation ($\bar{x}\pm \text{SD}$) and analyzed using SPSS 19.0 statistical software (SPSS Inc., USA). For variables in accordance with normal distribution, one-way ANOVA was performed followed by the LSD test. The nonparametric test was used to evaluate variables which were not in accordance with normal distribution. *P* < 0.05 was regarded as statistically significant.

## 3. Results

### 3.1. Both EA and MB Generate Therapeutic Effects on DSS-Induced Colitis Model Mice with Anxiety Behavior

Mice in the NG group had normal food and water intake, were active, and had dense and shiny pelts. Their feces appeared normal, and their perianal skin remained clean, while the MD group shown debility and anorexia; food intake was reduced, and the hairs appeared rough and less shiny. They reduced activity levels and were easily startled; they had increased stool frequency, bloody mucus was seen in the feces, the perianal skin was dirty with feces, and the DAI scores increased significantly. In summary, the manifestations of DSS colitis may include watery diarrhea, occult blood in stools, weight loss, decreased appetite, and decreased movements. After the treatment of EA and MB, mice displayed better food intake, responsiveness, activity levels, several symptoms such as mucopurulent stool, and diarrhea, and other severe parameters which have improved significantly. Therefore, the DAI score which has been used as a good index to evaluate the severity of colitis has decreased significantly [40] (Table 2).

**Figure 2:**
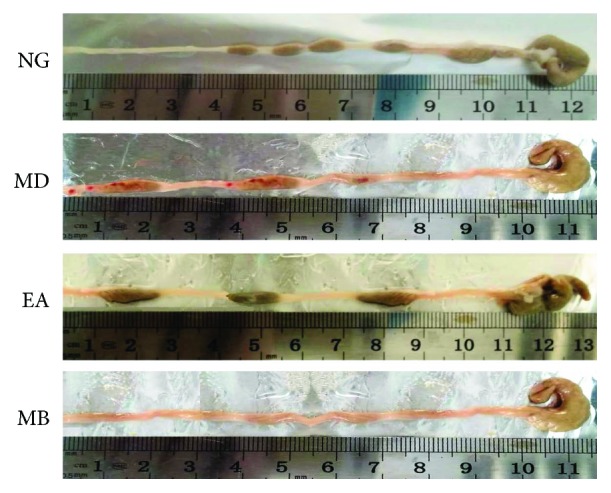
Appearance and morphology of the colon were observed in different groups. The NG showed normal intestinal appearance. The MD showed inflammatory intestinal appearance, and hyperemia, edema, and tissue necrosis were observed in distal colonic tissues. The EA and MB showed greatly improved appearance of the colon after receiving different treatments. NG: normal group; MD: model group; EA: electroacupuncture group; MB: moxibustion group.

**Figure 3:**
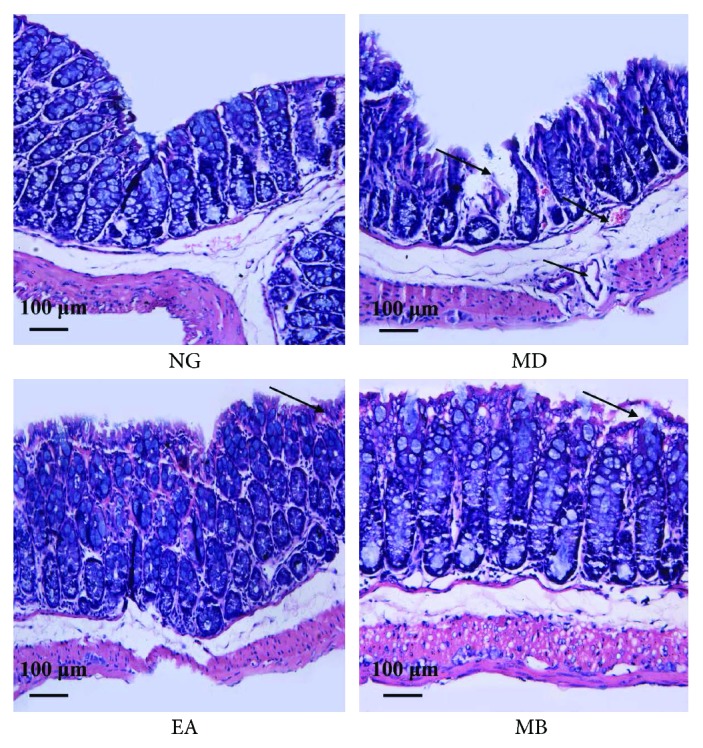
Pathological morphology of colon tissues was observed by hematoxylin-eosin staining. The NG showed continuous structure of mucosal epithelium and complete intestinal glands. Damaged mucosa, disordered glandular structure, and hyperemia were observed in the MD group. And the EA and MB showed greatly improved histopathological conditions after receiving EA and MB treatments. NG: normal group; MD: model group; EA: electroacupuncture group; MB: moxibustion group. Black arrow: representative features of colonic tissue (magnification: 200x, scale bar = 100 micron).

Histopathology analysis showed that, for the NG group, the intestinal mucosa was complete and continuous, the arrangement of gland was regular, the structure was clear, and there was no hyperemia, edema, and tissue necrosis; or inflammatory cell was soaked. In contrast, colonic tissues of mice from the MD group presented damaged mucosa and submucosae; disordered glandular structure and submucosae were congestive, edematous, and ulcerative; and inflammatory cell was visible. However, after the treatment of EA and MB, decreased inflammatory cell infiltration and improved colonic mucosa were seen. The arrangement of the mucosa was somewhat intact. The arrangement of the gland was relatively regular, and there were new epithelial cells on the ulcerations and only slight inflammatory cell in filtration. Among them, the recovery in the MD group was slightly superior to that in the EA group (Figures 2 and 3). The results suggested that EA and MB treatment can inhibit inflammatory cell infiltration under UC conditions and induce recovery of these ulcers in the colon tissue.

### 3.2. EA and MB Improve Anxiety Behavior in DSS-Induced Colitis Model Mice

**Figure 4:**
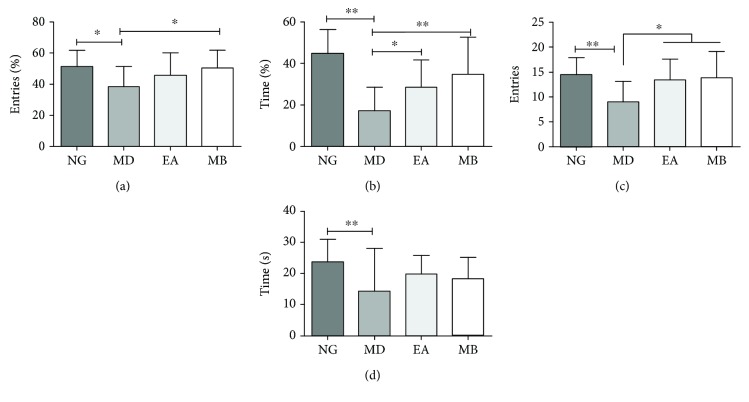
The anxiety-like behavior in elevated plus maze (EPM) and open field test (OFT). Percentage of the entries into the open arms (a) and time spent in the open arms (b) in the EPM test. The accumulative time of entering the central area (c) and spent in the central area (d) in the OFT. NG: normal group; MD: model group; EA: electroacupuncture group; MB: moxibustion group (^∗^*P* < 0.05 vs. MD group).

EPM and OFT tests were used to evaluate the effectiveness of EA and MB on improving the anxiety behavior of DSS-induced mice. For the EPM test, DSS-induced colitis mice displayed significantly fewer entries into the open arm (OE %) (Figure 4(a)) and spent significantly less time in the open arm (OT %) than the NG mice (Figure 4(b),*P* < 0.05). It was indicated that there is greater anxiety behavior in DSS-induced colitis model mice vs. the NG mice. However, compared with the MD group, OE% and OT% values were increased significantly after EA and MB treatment (*P* > 0.05) (Figures 4(a) and 4(b)). Similar findings were obtained in the OFT; the values of OFT have significantly increased after treatment (Figures 4(c) and 4(d)). These results suggested that EA and MB play an important role in improving and alleviating anxiety behavior in DSS-induced colitis model mice.

### 3.3. EA and MB Improve Levels of CRH and CORT in Serum of DSS-Induced Colitis Model Mice

**Figure 5:**
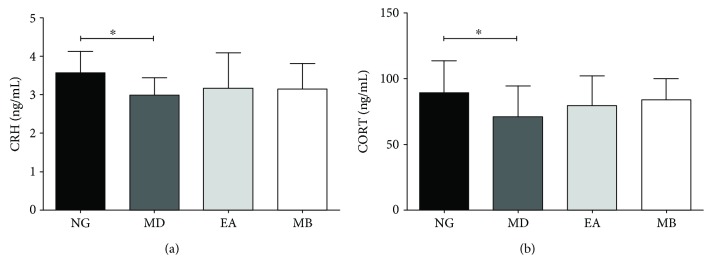
The CRH and CORT concentration in serum. The results showed that the concentration of CRH and CORT in the MD group significantly decreased (*P* < 0.05 vs. NG group). On the contrary, after EA or MB interventions, the concentrations of CRH and CORT were slightly increased. (a) The CRH concentration and (b) the CORT concentration. CRH: corticotropin-releasing hormone; CORT: cortisol. NG: normal group; MD: model group; EA: electroacupuncture group; MB: moxibustion group. *n* = 12/group (^∗^*P* < 0.05 vs. MD group).

In order to determine whether the effectiveness of EA and MB improvement alleviates anxiety in colitis model mice linked to the “HPA axis,” we had measured serum levels of CRH and CORT in the various groups by means of ELISA. Compared with the NG group, the serum levels of CRH and CORT in the MD group were decreased significantly (*P* < 0.05). Compared with the MD group, the serum levels of CRH and CORT after EA or MB treatment were increased (*P* > 0.05). However, the trends were not so significant since the *P* value is >0.05 (Figure 5).

### 3.4. EA and MB Improve Protein of CRH in the Colon from DSS-Induced Colitis Model Mice

**Figure 6:**
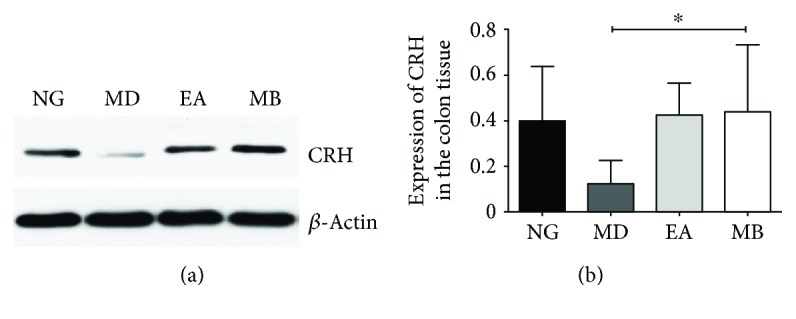
The protein expressions of CRH in the distal colon tissue. (a) The protein expression of CRH in the colon was evaluated by Western blot analysis. (b) Quantification of (a) (*n* = 12). The results showed that the intestinal CRH expression in the MD group was reduced (vs. NG group, *P* > 0.05), while it significantly enhanced by EA and MB treatment (vs. MD group). In addition, there was an obvious difference between the MD and the MB groups (^∗^*P* < 0.05). CRH: corticotropin-releasing hormone. NG: normal group; MD: model group; EA: electroacupuncture group; MB: moxibustion group.

In the EPM and OFT experiments, EA or MB effectively relieved anxiety behavior in DSS-induced colitis model mice. In order to determine whether this effect was achieved by regulating the protein expression of CRH in the peripheral colon, we performed further investigations by means of WB analysis. The results of WB had shown that the CRH protein expression of the distal colon in the MD group was significantly reduced, while, after EA and MB treatment, these two proteins have been significantly enhanced. In addition, the protein expression of CRH in the MB group was more than that in EA group (*P* < 0.05), which indicated the effectiveness of MB was a little bit better than that of EA. All the results suggested that EA and MB at the ST36 and CV4 acupoints could increase the CRH protein expression in the colon and effectively relieve anxiety behavior in DSS-induced colitis model mice (Figure 6).

### 3.5. The Positive Target Expression of CRH in the Hypothalamus

**Figure 7:**
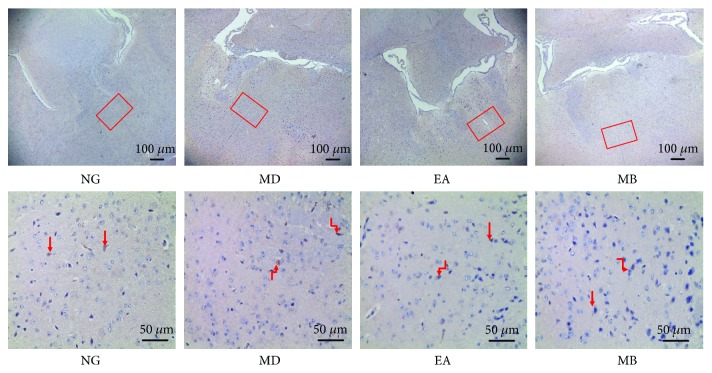
The expression of CRH in the hypothalamus was detected by the immunohistochemical method. The red square was the paraventricular nucleus (PVN), and red arrows were the positive cells of CRH in the PVN. NG: normal group; MD: model group; EA: electroacupuncture group; MB: moxibustion group. CRH: corticotropin-releasing hormone (magnifications—up: 40x, down: 400x, and scale bar = 50 micron).

**Figure 8:**
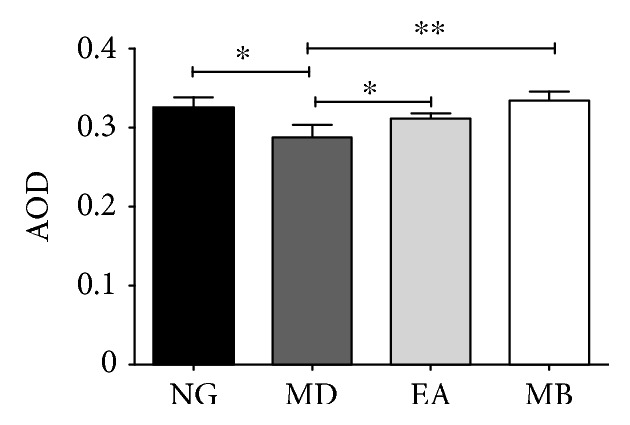
The average optical density (AOD) of CRH in the hypothalamus. The results (*n* = 8/group) showed that the AOD of hypothalamic CRH expression in the MD group was significantly reduced (vs. NG group, *P* < 0.05), but that in both the EA and the MB groups was significantly enhanced (vs. MD group; ^∗^*P* < 0.05 and ^∗∗^*P* < 0.01). CRH: corticotropin-releasing hormone. NG: normal group; MD: model group; EA: electroacupuncture group; MB: moxibustion group.

Although the above experiment suggested that CRH protein levels in the colon were involved in the modulation of DSS-induced colitis model mice by EA or MB, CRH levels in the central nervous system also played a role remaining unknown. Therefore, we performed further investigations by the immunohistochemical method, which measured the expression of CRH-positive cell (Figures 7 and 8) and protein in the hypothalamus (Figure 8). CRH was expressed in the paraventricular nucleus (PVN) as shown in Figures 7 and 8. The results show that the average optical density (AOD) of CRH expression of the hypothalamus in the MD group was significantly reduced (compared with that in the NG group, *P* < 0.05). However, after EA and MB treatment, these two groups' AOD of CRH expression has been significantly enhanced (compared with the MD group's, *P* < 0.05). Moreover, there is no obvious difference between the EA group and the MB group (*P* > 0.05). These results were suggested that EA and MB at the ST36 and CV4 acupoints could increase the CRH-positive cell expression in the hypothalamus to effectively relieve anxiety behavior in DSS-induced colitis model mice.

### 3.6. EA and MB Improve Protein of CRH in the Hypothalamus from DSS-Induced Colitis Model Mice

**Figure 9:**
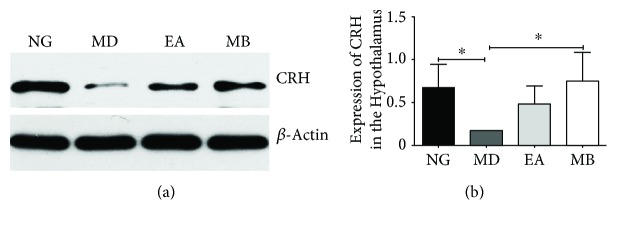
The expressions of CRH in the hypothalamus. (a) Western blot analysis of CRH in the hypothalamus. (b) Quantification of (a) (*n* = 6/group). The results showed that the hypothalamic CRH expression in the MD group was significantly reduced (vs. NG group, ^∗^*P* < 0.05), but that in both the EA and the MB groups was enhanced (vs. MD group), and there was obvious difference between MD and MB group (^∗^*P* < 0.05). CRH: corticotropin-releasing hormone. NG: normal group; MD: model group; EA: electroacupuncture group; MB: moxibustion group.

We measured the expression of CRH protein in the hypothalamus. Similarly, the CRH protein expression of the hypothalamus in the MD group was significantly reduced, while, after EA and MB treatment, these two proteins have been significantly elevated. Additionally, the protein expression of CRH in the MB group was more than that in the EA group (*P* < 0.05), which indicated the effect of MB was a little bit better than that of EA (Figure 9). All the results suggested that EA and MB at the ST36 and CV4 acupoints could increase the CRH protein expression in the colon to effectively relieve anxiety behavior in DSS-induced colitis model mice. There are changes in the expression of CRH from the peripheral colon to the central hypothalamus, which may be an important pathway of EA or MB antidepressant response in colitis model mice, but more evidence is needed to prove this specificity.

## 4. Discussion

Ulcerative colitis (UC) is a chronic inflammatory disease of the gastrointestinal tract, with increasing incidence and prevalence worldwide, affecting over 1.4 million Americans and accounting for an estimated $6 billion in direct healthcare costs [41, 42]. They are associated with considerable indirect costs in lost productivity and days absent from work. Up to a third of patients with UC may require surgical treatment of their disease, for refractoriness to medical therapy or cancer [43, 44]. High comorbidity is observed between UC and other diseases in which inflammation may be involved, including brain diseases such as cognitive impairment and anxiety. In recent years, people have paid attention to depression and anxiety in ulcerative colitis.

### 4.1. Relationship of Anxiety and UC

As observed in many other chronic diseases, psychological distress is highly prevalent in IBD [45]. A larger nested case-control study reported that higher rates of anxiety were found for those with IBD compared to those without IBD and were particularly severe during periods of active disease [46]. For CD patients, anxiety occurred 5 times more often than for controls, and for UC patients, anxiety occurred almost 4 times as often and depression twice as often as for controls [47]. For instance, Nahon et al. administered a questionnaire to 1663 patients with IBD and identified 11% of patients to be depressed and 41% to be anxious [48]. Further research found that the rate of anxiety and/or depression has been estimated at 29%–35% during periods of remission and as high as 80% for anxiety and 60% for depression during relapses. The most recent literature considers the nature of IBD and psychiatric disorders which include depression and anxiety. A Hungarian study using matched samples of tertiary clinic IBD patients and healthy controls found that the IBD patients had significantly higher levels of anxiety and depression symptoms than the healthy control group based on validated symptom measures [49]. On the other hand, animal and experimental evidences had indicated that ulcerative colitis-like inflammation also induces anxiety behavior in rats [50]. Similarly, Emge et al. [51] had tested the behavior of DSS-induced colitis model mice by the light-dark box test during active and remission phases of inflammation. The mice show marked anxiety behavior when active, while there was no significant difference between the MD and the NG group in the remission phase of inflammation. In this study, we employed well-established behavioral tests, EPM and OFT, to assess anxiety behavior, and the results showed that the EPM and OFT decreased score of DSS-induced colitis model mice was abnormally elevated, indicating the existence of anxiety in MD mice (Figure 4). It can be seen that both UC patients and UC animal model had anxiety and other negative emotions, and actively interfering with anxiety and other negative emotions may be an important way to improve the clinical efficacy of UC patients and their quality of life.

### 4.2. In the Context of the Co-Occurrence of Anxiety and UC, HPA Axis Is Considered to Contribute to the Risk

The HPA axis is a major component of the neuroendocrine system that controls reactions to stress and regulates many physiological processes, including digestion, immune response, mood and emotions, sexuality, and energy storage and expenditure. As a peptide hormone secreted from the paraventricular nucleus (PVN) of the hypothalamus, CRH initiates the stress response and subsequent release of ACTH and CORT and is associated with HPA axis dysfunction [52]. Besides, several studies have suggested that CRH is a major stress-related peptide in the HPA axis, which plays an important role in the occurrence of anxiety and eliciting anxiety symptoms [19–21]. It has been demonstrated that dysregulations of the CRH-HPA axis system may contribute to pathological anxiety [53, 54]. Some studies have confirmed that the excessive activation of the HPA axis with a mass secretion of CRH is an important biological basis for pathological anxiety [17, 55]. For instance, Dore et al. found that CRH receptor antagonist can fully block intracerebroventricular pituitary adenylate cyclase-activating polypeptide (PACAP) treatment-induced anxiety behavior in the EPM test and elevated intracranial self-stimulation thresholds [56]. Moreover, clinical studies have found that anxiety scores and the plasma CRH levels are significantly increased in patients with posttraumatic stress disorder [57]. It can be seen that the occurrence of anxiety behavior is closely related to the hyperactivity of the HPA axis. Animal experiments have also showed that CRH can activate the anti-inflammatory response of intestinal mucosa to maintain intestinal barrier integrity by regulating autophagy of the intestinal epithelial cells [58, 59]. It confirms [59] that CRHR2 receptor antagonists (Astressin2B) can block the TLR4/NF-*κ*B signaling pathway in DSS-induced colitis mice to reduce the levels of TNF-*α*, IL-6, and IL-1*β* in serum, thereby protecting the colon mucosa. Therefore, the CRH secreted by the HPA axis is directly or indirectly involved in intestinal mucosal immune abnormalities in UC. In the present study, we found that CRH expression in the plasma, colon, and hypothalamus of DSS-induced colitis mice was significantly reduced, which is inconsistent with the literature reports (Figures 5–8). One of the reasons may be due to the different responses of DSS-induced colitis in mice species selected for the experiment, in which the main manifestation was that the concentration of CRH with anti-inflammation decrease or this model was an acute UC model. In the acute inflammatory phase, the activity of the HPA axis is low, adrenal function is decreased, and the anti-inflammatory reaction is inhibited. Another reason is probably because the anti-inflammatory effect of CRH depends on CRHR2 which induces a proinflammatory effect during acute colitis [60] but confers protective activity during chronic inflammation [61]. Selective inhibition of CRHR2 signaling in experimental colitis mice could promote disease activity, destroy the impaired intestinal barrier, increase colonic epithelial cell apoptosis, and decrease epithelial cell proliferation [62]. Therefore, a further study should be focused on CRH and its receptor 2 (CRHR2) so as to clarify the underlying mechanism.

### 4.3. Treatment of Anxiety Behavior in DSS-Induced Colitis

Acupuncture and moxibustion, widely known as alternative medicine therapies, have been used in eastern countries for the treatment of various disorders, including mental disorders [63]. Although the mechanisms of acupuncture and moxibustion are not yet clear, several lines of evidence suggest that they can contribute to the maintenance of biochemical balance in the central nervous system and recovery of homeostasis in the body. This regulation is achieved through a series of biological changes produced after the acupuncture/moxibusiton signal activates the neuroendocrine system, which widely influences various systems, such as the digestive system [64, 65]. As the center of endocrine and autonomic nerves, the hypothalamus is indispensable in physiological and pathological processes, such as the HPA axis which is vital for adjusting various stresses. Few studies have shown that acupuncture is effective on regulating hypothalamic CRH secretion in stress-related diseases, caused by imbalance of the HPA axis [66]. In addition, some literatures report that CRH is an important indicator to assess acupuncture in attenuating chronic stress-induced symptoms [18, 30]. Park et al. [67] demonstrated that acupuncture at ST36 could ameliorate restraint stress-induced anxiety by modulating plasma CORT and tyrosine hydroxylase levels in rats.

Interestingly, our results showed that the levels of CRH and CORT were decreased significantly in the DSS-induced colitis model mice with anxiety behavior. However, some previous studies suggested that CRH [16] and CORT [68] were relatively highly secreted in the stress-induced anxiety model and irritable bowel syndrome (IBS) model [18] with a high anxiety state. These results suggested that the secretion levels of CRH and CORT may vary in different primary diseases. For instance, the release of CRH or CORT is mainly influenced by stress in the studies mentioned above, while the primary disease is inflammation in this study. Besides, the phase of disease, the level and activity of CRHR2 (as we mentioned in previous discussion), and the circadian cortisol cycle should be considered too. Therefore, more evidence is needed to define the role of CRH and CORT in inflammatory disease with anxiety symptoms. Anyway, from the present study, the results indicate that regulating CRH and CORT feedback may be an important way of EA and MB to ameliorate anxiety behavior in DSS-induced colitis.

## 5. Conclusions

In summary, the cause and treatment of illness are based on a holistic understanding of the human body. The HPA axis is a major component of the neuroendocrine system that is critical for maintaining internal homeostasis. This study proved that both EA and MB can ameliorate anxiety behavior in DSS-induced colitis and the effects are partially from improving the balance of the HPA axis. In addition, our study indicated whether MB has better efficacy and EA needs further study.

## Figures and Tables

**Table 1:** Ulcerative colitis disease activity index.

Body weight loss (%)	Characteristics of feces	Fecal occult blood/gross fecal blood	Scoring
0	Normal	Normal	0
1–5			1
6–10	Loose fecal	Fecal occult blood	2
11–15			3
>16	Watery feces	Naked eye bloody feces	4

**Table 2:** Comparison of DAI in each group (mean ± SD).

Group	*n*	Pretreatment	Posttreatment
NG	12	—	—
MD	12	2.64 ± 0.48	2.63 ± 0.51
EA	12	2.65 ± 0.45	1.12 ± 0.57^∗^
MB	12	2.63 ± 0.49	1.09 ± 0.46^∗^

NG: normal group; MD: model group; EA: electroacupuncture group; MB: moxibustion group (^∗^*P* < 0.05 vs. the model group).

## Data Availability

The data used to support the findings of this study are available from the corresponding author upon request.
